# A Review: The Fate of Bacteriocins in the Human Gastro-Intestinal Tract: Do They Cross the Gut–Blood Barrier?

**DOI:** 10.3389/fmicb.2018.02297

**Published:** 2018-09-28

**Authors:** Leon M. T. Dicks, Leané Dreyer, Carine Smith, Anton D. van Staden

**Affiliations:** ^1^Department of Microbiology, Stellenbosch University, Stellenbosch, South Africa; ^2^Department of Physiological Sciences, Stellenbosch University, Stellenbosch, South Africa

**Keywords:** bacteriocins, antibiotics, microbiota, gut–blood barrier, probiotics

## Abstract

The intestinal barrier, consisting of the vascular endothelium, epithelial cell lining, and mucus layer, covers a surface of about 400 m^2^. The integrity of the gut wall is sustained by transcellular proteins forming tight junctions between the epithelial cells. Protected by three layers of mucin, the gut wall forms a non-permeable barrier, keeping digestive enzymes and microorganisms within the luminal space, separate from the blood stream. Microorganisms colonizing the gut may produce bacteriocins in an attempt to outcompete pathogens. Production of bacteriocins in a harsh and complex environment such as the gastro-intestinal tract (GIT) may be below minimal inhibitory concentration (MIC) levels. At such low levels, the stability of bacteriocins may be compromised. Despite this, most bacteria in the gut have the ability to produce bacteriocins, distributed throughout the GIT. With most antimicrobial studies being performed *in vitro*, we know little about the migration of bacteriocins across epithelial barriers. The behavior of bacteriocins in the GIT is studied *ex vivo*, using models, flow cells, or membranes resembling the gut wall. Furthermore, little is known about the effect bacteriocins have on the immune system. It is generally believed that the peptides will be destroyed by macrophages once they cross the gut wall. Studies done on the survival of neurotherapeutic peptides and their crossing of the brain–blood barrier, along with other studies on small peptides intravenously injected, may provide some answers. In this review, the stability of bacteriocins in the GIT, their effect on gut epithelial cells, and their ability to cross epithelial cells are discussed. These are important questions to address in the light of recent papers advocating the use of bacteriocins as possible alternatives to, or used in combination with, antibiotics.

## Introduction

Bacteriocins are ribosomally synthesized peptides with antibacterial activity (Cavera et al., 2015). Bacteriocins can be post-translationally modified (PTM) or non-modified and are grouped into different classes (Cotter et al., 2005; Heng et al., 2006; Arnison et al., 2013; Alvarez-Sieiro et al., 2016). For example, lantibiotics are modified bacteriocins and are grouped in class I, they are membrane-active peptides with thioether-containing amino acids lanthionine and β-methyllanthionine. According to the classification scheme proposed by Heng et al. (2006), class I is subdivided into three types of lantibiotics (types A–C). Linear lantibiotics were classified as type A, globular lantibiotics as type B, and multi-component lantibiotics, requiring two or more modified peptides for bioactivity as type C. Subsequent classification schemes for class I have been updated, including changes to the classification of lantibiotics and inclusion of other PTM bacteriocins (Arnison et al., 2013; Alvarez-Sieiro et al., 2016). An extensive review by Arnison et al. (2013) expanded on the nomenclature and classification of modified bacteriocins with the proposed recommendations changing and expanding on previous classification schemes. For example, lanthipeptides (including lantibiotics) are separated into four classes based on modification machinery and using this classification system for lanthipeptides each class can further be grouped based on amino acid sequences of the modified peptides (Cotter et al., 2005; Arnison et al., 2013; Van Staden, 2015). Additionally, the review by Arnison et al. (2013) also expands on the classification and nomenclature of non-lantibiotic/lanthipeptidemodified peptides. As reviewed by Alvarez-Sieiro et al. (2016), the unmodified, membrane active, heat stable bacteriocins are grouped in class II, with four subclasses based on structural differences and their mode of action. Anti-listeria, pediocin-like peptides, are grouped in subclass IIa and bacteriocins that require two or more peptides for activity in subclass IIb. Leaderless bacteriocins, without an N-terminal leader peptide, are grouped in class IIc and unmodified bacteriocins that are not pediocin-like or multicomponent bacteriocins are grouped in subclass IId. Large heat liable bacteriocins are grouped in class III, subdivided into IIIa (bacteriolysins) and IIIb (non-lytic proteins).

Classification schemes for bacteriocins are constantly evolving to accommodate the increase in complexity and diversity of these peptides. Furthermore, with increased understanding of how bacteriocins function and identification of novel bacteriocins the systems in place to group them will need to adapt and change accordingly.

Most antimicrobial peptides are positively charged and smaller than 10 kDa (with the exception of the class III bacteriocins). Their small size, charge, and variation in hydrophobic and hydrophilic properties allow them to adhere to microbial cells and penetrate phospholipid membranes (Izadpanah and Gallo, 2005). Bacteriocins adhere to target organisms via “docking molecules.” Lipid II, for example, serves as the docking molecule for several lantibiotics (mostly nisin-like). Mannose phosphotransferase proteins IIC/D serves as docking molecule for class IIa bacteriocins (Diep et al., 2007). The mode of action is either pore formation, thus destabilizing the proton motive force, or inhibition of DNA-, RNA-, and protein-synthesis (Nishie et al., 2012; Cotter et al., 2013). Some bacteriocins exhibit a much broader spectrum of activity and may extend beyond the borders of bacteria to include protozoa, yeast, fungi, viruses, and eukaryotic cells (e.g., cancer cells and spermatozoa; Reddy et al., 2004; Drider et al., 2016; Chikindas et al., 2017).

In their natural environment, bacteria produce bacteriocins to compete against other bacteria for nutrients. Complex environments with changes in growth conditions, nutrients, pH, water activity (Aw), and temperatures will have an effect on bacterial cell numbers, their metabolic activity, and bacteriocin production. In the gastro-intestinal tract (GIT), changes in food particles and fluctuation in spices, additives, salts, bile, digestive enzymes, etc., may have a negative impact on cell growth and bacteriocin production. Once secreted by the producer cells, the activity of bacteriocins may be affected by their ability to adhere to food particles and diffuse through the digesta, but also their stability at different pH values, resistance to digestive enzymes and proteolytic enzymes, and chemical interactions with particles and microbial cells in the GIT. On the other hand, an increase in salt or EDTA levels may increase the sensitivity of Gram-negative bacteria to bacteriocins (Dykes and Hastings, 1998; Bouttefroy and Milliere, 2000; Vignolo et al., 2000). It is thus extremely difficult to visualize the functioning of bacteriocins in the GIT. The production of nisin A, for example, is regulated by a protein pheromone, via a two-component signal transduction system similar to quorum-sensing systems. In a fermentor, the addition of nisin to a culture stimulates the expression of nisin. This may very well be the situation in the GIT. An increase in bacteriocin levels, e.g., when adhered to lipids in the mucus layer, may have an effect on the expression of genes encoding bacteriocin production. These are intriguing questions and need to be studied, as it may affect the levels at which bacteriocins accumulate in the GIT. If concentrated in localized areas of the GIT, bacteriocins may cross the epithelial barrier easier.

A recent study, conducted by Drissi et al. (2015), explored the role bacteriocins may have in the GIT. In a genome mining project, the authors retrieved 641 genomes (307 whole genomes and 334 draft genomes) from microorganisms in the human gut. The genomes represented 199 bacterial genera, including *Lactobacillus*, *Streptococcus*, *Clostridium*, and *Bacillus.* A bidirectional protein BLAST, compared to bacteriocin sequences listed in the BUR database, revealed that 317 of the genomes encoded putative bacteriocins of classes I (44%), II (38.6%), and III (17.3%). This supports the hypothesis that bacteriocins are widespread across the GIT. Of the 317 putative bacteriocins, 175 were from Firmicutes (which includes LAB), 79 from Proteobacteria, 34 from Bacteroidetes, and 25 from Actinobacteria. The high number of bacteriocins being (hypothetically) produced by Proteobacteria may explain why they are so persistent and virulent. The study also suggested that bacteriocins produced by gut bacteria are generally smaller in size and differ in amino acid composition compared to most other bacteriocins. Furthermore, these (putative) bacteriocins contained less aspartic acid, leucine, arginine, and glutamic acid, but more lysine and methionine. Based on their α-helical structure, charge, and hydrophobicity, they possibly have a broad spectrum of antimicrobial activity (Dathe and Wieprecht, 1999; Giangaspero et al., 2001; Zelezetsky and Tossi, 2006). Considering these findings, the bacteriocins produced by gut bacteria, especially Firmicutes and Proteobacteria, may render them a competitive advantage over other bacteria in the GIT (Schuijt et al., 2013). Drissi et al. (2015) speculated that bacteriocins in the GIT may have low levels of antimicrobial activity and may thus not have such a drastic effect on microbial populations. This makes sense, as it supports the existence of a large variation of gut bacteria, thus a balanced population. If bacteriocins play a lesser role in population dynamics, they may have a greater role to play in quorum sensing, or possibly in host immune modulation.

## From Food Preservatives to Infection Fighters

A few decades ago most research groups studied bacteriocins of LAB for their food preservation properties (Cotter et al., 2005). Since most LAB have generally regarded as safe (GRAS) status, their bacteriocins are regarded safe by the US Food and Drug Administration (Chen and Hoover, 2003; Montville and Chikindas, 2013). However, despite all the research on bacteriocins, only a few have been approved as food preservatives. Of these, the lantibiotic nisin, produced by *Lactococcus lactis* subsp. *lactis*, is the best studied and is used to preserve a number of foods, mostly dairy products and canned vegetables (Johnson et al., 2017). Bacteriocins produced by *Pediococcus acidilactici* have also been added to fresh milk to prevent the growth of *Listeria* spp. (Johnson et al., 2017). Several bacteriocinogenic LAB have been used as starter cultures, e.g., the fermentation of sausages and cheese (e.g., *P. acidilactici* and *Lactobacillus plantarum*) and vacuum-packed beef, e.g., *Leuconostoc gelidum* (Johnson et al., 2017).

During the last decade, an increasing number of papers were published suggesting that bacteriocins may be used in the prevention or treatment of bacterial infections (**Table 1**). However, despite these evidences, only nisin has been approved for use in oral/topical use. Other peptide antibiotics approved for clinical use include gramicidin, daptomycin, vancomycin, and polymyxin, which are non-ribosomally synthesized and thus not classified as bacteriocins. The diversity of bacteriocin-producing bacteria and the wealth of literature supporting the efficacy of bacteriocins *in vivo* render them ideal candidates for treatment of bacterial infections. The development of bacteriocins for clinical applications is, however, hampered by production costs, stability/solubility issues, and possible cytotoxic effects. These shortcomings can be overcome. Nisin and lacticin 3147, for instance, can be produced cost effectively at large scale with optimization of fermentation techniques and the use of heterologous expression systems. Companies such as Novacta Biosystems and Oragenics are developing large-scale fermentation and recovery processes for lantibiotics. Another company spearheading the development of lanthipeptides is LanthioPharma that focus on the discovery and development of lanthipeptide-based drugs for various clinical (other than antimicrobial) applications. By using lanthipeptides, LanthioPharma are developing novel peptides, and incorporating lanthionines into existing peptides (e.g., apelin), that are more stable and resistant to protease degradation. Bacteriocins can also be delivered via bacteriocin-producing bacteria. Two strains are being commercialized for their ability to produce bacteriocins (BLIS K12^TM^ and BLIS M18^TM^). Many probiotic formulations contain strains that produce bacteriocins; however, they are not marketed as such. Probiotic bacteria may serve as a method of delivering bacteriocins to the GIT, in that the cells protect the peptides against acids and proteases in the stomach (Marteau and Shanahan, 2003).

**Table 1:** Examples of bacteriocins with bioactivity and their potential applications.

Bacteriocin	Producer strain	Tested	Bioactivity	Potential Applications	References
Nisin	*Lactococcus lactis*	*In vitro*	Antimicrobial agent	Skin infections, GIT infections, respiratory tract infections, immune modulation, gingivitis, prosthetic implant infections, cancer treatment, wound healing	Howell et al., 1993; Aranha et al., 2004; Bartoloni et al., 2004; De Kwaadsteniet et al., 2009; Begde et al., 2011; Van Staden et al., 2011, 2012, 2015; Joo et al., 2012; Campion et al., 2013; Heunis et al., 2013; Kindrachuk et al., 2013; Kamarajan et al., 2015; Van Staden, 2015
		*In vivo*	Anticancer agent		
		TC^#^	Immune modulation		
Gallidermin/epidermin	*S. gallinarum/S. epidermidis*	*In vitro*	Antimicrobial agent	Skin infections, prosthetic implant infections	Bonelli et al., 2006; Kindrachuk et al., 2013; Bengtsson et al., 2018
		TC^#^			
Mersacidin	*Bacillus amyloliquefaciens*	*In vitro*	Antimicrobial agent	Skin infections	Chatterjee et al., 1992; Kruszewska et al., 2004
		*In vivo*			
		TC^#^			
Duramycin	*Streptomyces cinnamoneus*	*In vitro*	Antimicrobial agent	Atherosclerosis treatment, cystic fibrosis treatment, immune modulation	Märki et al., 1991; Iwamoto et al., 2007; Oliynyk et al., 2010; Zhao, 2011; Audi et al., 2012; Hasim et al., 2018
		*In vivo*	Immune modulation		
			Ion channel modulation		
Lacticin 3147	*Lactococcus lactis*	*In vitro*	Antimicrobial agent	Skin infections, GIT infections, mycobacterial infections	Gardiner et al., 2007; Rea et al., 2007; Piper et al., 2009, 2012; Carroll et al., 2010
		*In vivo*			
Peptide ST4SA	*Enterococcus mundtii*	*In vitro*	Antimicrobial agent	GIT infections	Knoetze et al., 2008; Dreyer, 2018; Van Zyl, 2018
		*In vivo*			
		TC^#^			
Plantaricin 423	*Lactobacillus plantarum*	*In vitro*	Antimicrobial agent	GIT infections	van Reenen et al., 1998; Dreyer, 2018; Van Zyl, 2018
		*In vivo*			
		TC^#^			
Piscicolin 126	*Carnobacterium piscicola*	*In vitro*	Antimicrobial agent	GIT infections	Jack et al., 1996; Ingham et al., 2003
		*In vivo*			
Pediocin PA-1	*Pediococcus acidilactici*	*In vitro*	Antimicrobial agent	GIT infections	Cintas et al., 1998; Dabour et al., 2009
		*In vivo*			
Divercin V41	*Carnobacterium divergens*	*In vitro*	Antimicrobial agent	GIT infections	Rihakova et al., 2009, 2010
		*In vivo*			
Bac Abp118	*Lactobacillus salivarius*	*In vitro*	Antimicrobial agent	GIT infections	Flynn et al., 2002; Corr et al., 2007; Riboulet-Bisson et al., 2012
		*In vivo*			
Plantaricin A	*Lactobacillus plantarum*	*In vitro*	Antimicrobial agent	Cancer treatment, immune modulation, wound healing	Hauge et al., 1998; Sand et al., 2010, 2013; Pinto et al., 2011; Marzani et al., 2012
		TC^#^	Anticancer agent Cell migration/proliferation		
			Immune modulation		


*^#^Tissue culture.*

The mode of action of bacteriocins is remarkably different from conventional antibiotics and the machinery used by pathogens to develop resistance should be different. With this in mind, bacteriocins may be considered as “new age infection fighters.”

## The Need for Alternative Antimicrobials

The discovery of penicillin in 1928 by Alexander Fleming was a life-changing event in the history of medicine (Tan and Tatsumura, 2015). However, the first report of pathogenic *Staphylococcus aureus* strains resistant to penicillin was published in the early 1940s (Chambers and Deleo, 2009). This urged scientists to search for alternative antibiotics and sulfonamides that were described in 1935. More than 20 classes of antibiotics were described between 1940 and 1962 (Coates et al., 2002; Powers, 2004), referred to as the “golden age” of antibiotics. Looking back, it seems as if everyone was at peace with the antibiotics on the market. No new classes of antibiotics were developed between 1968 and 1998. In fact, the few antibiotics developed up to 1960 had to suffice in curing all types of bacterial infections for the next 50 years (Coates et al., 2011). In hindsight, this was an unreasonable expectation, keeping in mind the rate at which antibiotics develop resistance (Bax et al., 1998). Infections caused by Gram-negative bacteria are becoming increasingly difficult to treat, as many strains produce metallo-β-lactamase that neutralize carbapenems (Kumarasamy et al., 2010). With the current rate at which bacteria develop resistance, we may need more than 20 new classes of antibiotics to last the next 50 years, i.e., treat infections up to 2060. Nowadays, most of the deaths caused by *S. aureus* are due to methicillin-resistant strains (MRSA; Klein et al., 2007).

*Clostridium difficile* may be added to the list of most feared pathogens, the so-called ESKAPE group (*Enterococcus faecium*, *S. aureus*, *Klebsiella pneumoniae*, *Acinetobacter baumanii*, *Pseudomonas aeruginosa*, and *Enterobacter* species). Infections caused by *C. difficile* increased dramatically over the last decade, especially in patients with irritable bowel disease (IBD; Nguyen et al., 2008). Severe cases of CDI are treated with oral metronidazole (250–500 mg four times a day for 10–14 days), or oral vancomycin (125–500 mg four times a day for 10–14 days). Metronidazole is often administered intravenously, in doses of 500 mg four times daily (Persky and Brandt, 2000). Although metronidazole is the antibiotic of choice, failure rates of 22–38% have been reported and many strains have developed resistance (Miller et al., 2010, 2011).

The alarming rate at which strains become resistant is understandable, considering that antibiotics are among the most commonly prescribed drugs (Centers for Disease Control and Prevention [CDC], 2013). As many as 50% of the prescribed antibiotics are either not required, or are not effective in treating the infection. Despite this, doctors continue to prescribe antibiotics. In the United States, more than five prescriptions are written each year for every six patients (Truter, 2015). This leads to the natural selection of bacteria resistant to not one, but several antibiotics (Thomsen, 2016). Genes encoding resistance to antibiotics is shared among pathogens, often across species borders, through horizontal gene transfer. The World Health Organization (WHO) has already declared antibiotic resistance a global crisis, worse than the AIDS epidemic. Antimicrobial resistance to tuberculosis, hospital acquired infections, and common bacterial diseases is increasing mortality rates drastically. At least two million individuals in the United States contract serious antibiotic-resistant bacterial infections each year (O’Neill, 2014). Approximately 23,000 people die each year due to infections caused by antibiotic-resistant bacteria.

The peptide antibiotic vancomycin remains one of the most successful in the treatment of infections caused by Gram-positive bacteria. The reason is that vancomycin resistance would require changes in multiple steps in the peptidoglycan pathway. Similar to vancomycin, lanthipeptides such as nisin also targets a cell wall component, in this case lipid II. The antibacterial activity of antibiotics may be increased by combined use with a combination of bacteriocins (Bastos et al., 2015; Cavera et al., 2015). The most effective would be bacteriocins with different modes of action, preferably from different classes, to rule out the possibility of strains developing cross resistance (resistance to more than one bacteriocin). Further research is required to bioengineer bacteriocins with unique target sites and different modes of activity.

Similar to antibiotics, strains treated with bacteriocins may develop resistance (Arthur et al., 2014; Bastos et al., 2015). Mechanisms involved in bacteriocin resistance are either acquired or innate (Collins et al., 2012; Bastos et al., 2015). Innate resistance may develop by mimicking the bacteriocin-producing strain’s immunity system, degrading the bacteriocin, or adapting to changes in the bacterial cell-envelope and growth conditions (Bastos et al., 2015). Immunity mimicking is caused by non-bacteriocin-producing strains with genes homologous to the immunity genes of bacteriocin-producing strains. Expression of the homologous genes confers protection against the bacteriocin. Certain bacteria produce enzymes that degrade bacteriocins, e.g., nisinase produced by *Bacillus cereus* and *Paenibacillus polymyxa* (Jarvis, 1967). Mutations in genes coding for proteins involved cell wall structure lead to changes in cell surface charge, preventing the bacteriocin from binding to the cell. Stationary phase cells of *Listeria monocytogenes* 412 were more resistant to nisin and pediocin than cells in their exponential phase (Jydegaard et al., 2000). This is attributed to the fact that cells in stationary phase are more adaptable to stress conditions such as high or low osmotic concentrations, acidic conditions, and heat shock (Hu and Coates, 2012). Unlike innate resistance, the properties associated with acquired resistance are only found in certain strains of a species (Bastos et al., 2015). The mechanisms responsible for resistance vary greatly among strains and species. Acquired resistance results from gene mutations or horizontal gene transfer via transformation, conjugation, or transduction (Collins et al., 2012), altering the cell wall, cell membrane, receptors, and transport systems.

It is evident that we need novel antimicrobial compounds to treat bacterial infections. Oxazolidinone (linezolid by Pfizer) and cyclic lipopeptide (daptomycin by Cubist), with activity against Gram-positive bacteria, including MRSA, are two of the most recent antibiotics released into the market (Coates et al., 2011). There may be a number of yet to be published antibiotics that are currently in preclinical development, but the overall conclusion is that we are heading for a disaster if antibiotics with broader antimicrobial activity are not developed in the next few years. The rate at which novel antibiotics are being developed is just not sufficient to control bacterial infections. We need to focus our efforts in developing antibiotics that target complex bacterial systems, such as cell membranes. Bacteriocins may be an alternative to antibiotics.

## Structure of the Gut-Blood Barrier

**FIGURE 1:**
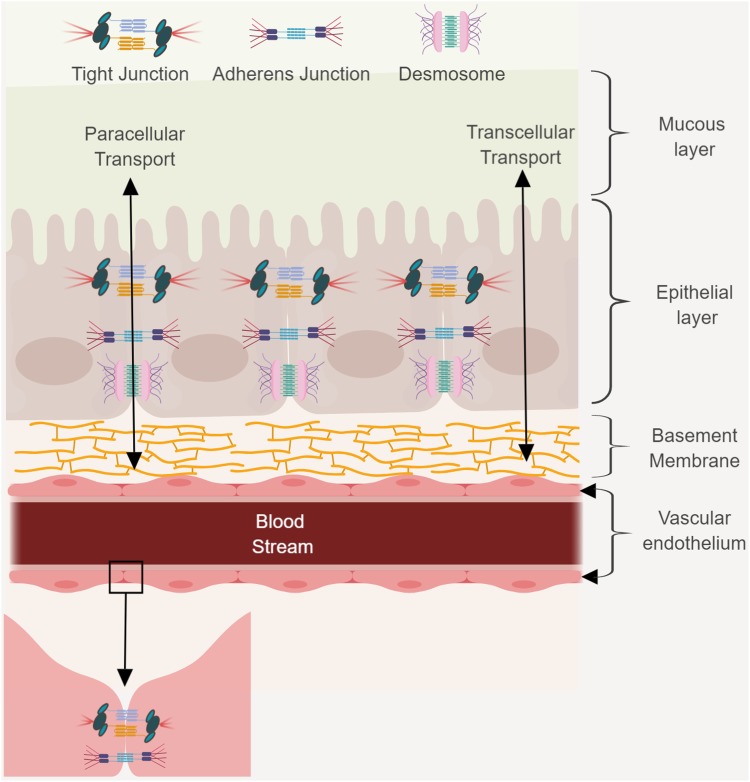
The GBB consists of a mucus layer, a monolayer of epithelial cells, and a monolayer of endothelial cells that line blood vessels. This barrier protects the host by preventing passage of harmful compounds or pathogens from the gut lumen to the bloodstream. Transcellular and paracellular transport and junctional complexes and desmosomes are indicated. Figure created in biorender (http://biorender.io).

The GBB is an intricate system, consisting of multiple layers (**Figure 1**). It plays an important role in maintaining homeostasis between the blood stream and GIT, and regulates the absorption of water, electrolytes, and nutrients from the gut lumen (Kelly et al., 2015). The intestinal barrier also serves as a protective barrier by preventing pathogenic microorganisms and luminal toxins from entering the blood stream (Farhadi et al., 2003). The mucus layer (consisting of large, highly glycosylated proteins), covering the epithelium, protects villi from physical friction caused by luminal content, and contact with toxins and bacteria (Farhadi et al., 2003). It forms an important diffusion barrier, restricting the movement of molecules and pathogens. Disruption of the intestinal mucus layer, or suppression of mucus production, may lead to hyper permeability.

The epithelial layer is a single layer of epithelial cells that line the gut lumen and are connected by desmosomes, tight junctions (TJs) and adherens junctions (AJs). TJs and AJs use transcellular proteins to connect to the actin cytoskeleton. The cytoskeleton is crucial for paracellular transport. The TJs are distributed across the gastro-intestinal membrane and the number of proteins varies between the small intestine, large intestine, villi, and crypts. The TJs control water and ion permeability, and the absorption of proteins and bacterial antigens. Intestinal epithelial cells (IECs) play an important role in the absorption of nutrients. The epithelium mediates selective permeability by transcellular and paracellular pathways. Lipophilic and small hydrophilic molecules pass through the barrier transcellularly, while larger hydrophilic molecules pass the barrier paracellularly. During transcellular permeability, solutes are transported through the epithelial cells. This is regulated by selective transporters for amino acids, short chain fatty acids, electrolytes, and sugars. During paracellular permeability, solutes are transported in spaces between epithelial cells. This is regulated by intercellular complexes present at the apical–lateral membrane junction. Amino acids and vitamins are transferred by means of active transport.

Nutrients, bacteria, and molecules that transverse the IEC to gain access to the blood stream first need to cross the vascular endothelium. Endothelial cells (ECs) line the interior surface of blood vessels and lymphatic vessels and form the endothelium (Michiels, 2003). ECs that make up the endothelium are connected to each other by TJs, AJs, and gap junctions. The endothelium is extremely important as it forms a selective barrier for the movement of molecules between blood and tissue. The existence of an additional barrier known as the gut–vascular barrier (GVB) that restricts the size and type of molecules translocating across the ECs has also been proposed (Spadoni et al., 2015). To evaluate the presence of this barrier, mice were injected with different molecular sizes of fluorescein isothiocyanate (FITC)-dextran and examined the intestine for any dye leakage. A molecule of 4 kDa had the ability to move through the endothelial barrier, whereas a molecule of 70 kDa could not. However, infection with *Salmonella typhimurium* could disrupt the GVB, resulting in translocation of the 70 kDa FITC-dextran (Spadoni et al., 2015).

Disruption of the epithelial and/or endothelial (GVB included) layers results in permeability of these selective barriers, which in-turn can lead to dysbiosis and subsequent disease states (e.g., irritable bowel syndrome and celiac disease). It is therefore crucial to maintain the integrity of the intestinal barrier, which prevents the crossing of microbes through the gut–blood barrier (GBB). However, the large body of literature in support of a gut microbiota–host interaction suggests that even in the absence of pathological increases in permeability of the barrier, some degree of permeability exist that allows for GBB crossing of microbial secretory products and/or metabolites – of which bacteriocins may form part. Before reviewing the relevant evidence specific to bacteriocins, it is relevant to consider how microbiota in general interacts with the GBB. In this context, there are two main topics to consider: first, how the microbiota enhances GBB integrity and second, how microbiota achieves signaling to modulate host health from within the gut as well as across the GBB.

## The Effect of GUT Microbiota on GBB Integrity

Over the past few years, the importance of gut microbiota in health and disease has caught significant attention. The manner in which microbiota interacts with the GBB and affect its permeability may have far reaching effects and may even lead to dysbiosis. To minimize the risk of disease, it is important to maintain a healthy gut microbiota. Furthermore, infections targeting the GIT can cause dysbiosis in the microbiota which is further exacerbated by the use of antibiotics. Additionally, inflammation of the GIT can also result in dysbiosis of this complex system (Buttó and Haller, 2016). Maintaining homeostasis within the GIT is of utmost importance as disturbances in the natural microbiota and intestinal barrier has been associated with several diseases such as such as IBD, diabetes, Alzheimer’s and stress related behaviors (Foster and McVey Neufeld, 2013; Simrén et al., 2013, Konstantinov et al., 2008; Jiang et al., 2017; Li et al., 2017).

Several examples of probiotic bacteria modulating the immune system and improving the GBB have been reported. Probiotic strains of *Bifidobacterium*, *Lactobacillus*, and *Streptococcus* used in a post-infectious IBD model, suppressed the expression of pro-inflammatory cytokines IL-6 and IL-17, and stimulated the expression of TJ proteins (claudin-1 and occluding), leading to enhanced barrier stability (Wang and Kasper, 2014). *Lactobacillus rhamnosus* GG decreased inflammation in an IL-10 receptor-dependent manner in an immature murine colon (Mirpuri et al., 2012). The increase in expression of the IL-10 receptor was also associated with reductions in TNF-α and MIP-2, both of which are pro-inflammatory. *Bifidobacterium* decreased production of IL-6 and TNFα in Caco-2 monolayers stimulated with LPS. *Bifidobacterium* also suppressed the expression of zonulin, responsible for dismantling TJs. This modulated the permeability of epithelium cells in Caco-2 monolayers, while upregulating the expression of occluding, claudin-2 and ZO-1 (Ling et al., 2016). The stabilizing effect *Bifidobacterium* has on Caco-2 monolayers can be translated to the *in vivo* environment of the gut (Ling et al., 2016). Probiotic *Streptococcus* and *Lactobacillus* strains reversed the negative effects observed with epithelial cell lines caused by entero-invasive *Escherichia coli* (Resta-Lenert and Barrett, 2003). Probiotic strains of *Lactobacillus acidophilus* and *Streptococcus thermophilus* increased trans-epithelial resistance with pre-treated monolayers and protected the cells from damage caused by *E. coli.* Probiotic administration was also associated with phosphorylation of cytoskeletal (actin and actinin) and TJ proteins (ZO-1 and occludin), providing stability to TJs. Furthermore, *Lactobacillus* species are also capable of stabilizing AJs through the increased expression of E-cadherin, as well as strengthening the E-cadherin/β-catenin complex through enhanced phosphorylation of β-catenin (Hummel et al., 2012). These studies and others illustrated how probiotics can modulate the host immune system and reduce inflammation. Enhancement of AJs and TJs maintains the intestinal barrier, providing protection against damage and preserving the integrity of the GBB.

Extracellular proteins secreted by *Bifidobacterium breve* C50 interacted with TLR-2 on the surface of immature DCs and induced a number of functional and physiological changes. Some of the effects included prolonged survival of DCs, earlier maturation of DCs, and an increase in IL-10 and IL-12 production (Hoarau et al., 2008). Proteins secreted by probiotic lactobacilli are involved in maintenance of the mucosal barrier, mainly through MAPK-dependent mechanisms (Schlee et al., 2008). Proteinaceous compounds secreted by *L. acidophilus* PZ 1138, *Lactobacillus fermentum* PZ 1162, and *Lactobacillus paracasei* subsp. *paracasei* LMG P-17806 stimulated the production of b-defensin 2 (hBD2) in human epithelial cells. The signal of these proteins was transduced to the nucleus through the MAPKs ERK, p38, and c-Jun terminal kinase (JNK). Synthesis of hBD2 increased through modulation of nuclear factor kB (NF-kB) and the activator protein 1 (AP-1). This resulted in increased IL-8 production (Schlee et al., 2008). Two from *L. rhamnosus* GG (NPSRQERR and PDENK) were antimicrobial toward *E. coli* EAEC 042, *Salmonella enterica* serovar Typhimurium and *S. aureus* (Lu et al., 2009).

Probiotic strains of *Bifidobacterium*, *Lactobacillus*, and *Streptococcus* suppressed the expression of pro-inflammatory cytokines IL-6 and IL-7, and stimulated the expression of TJ proteins, leading to enhanced barrier stability. *L. rhamnosus* GG interacted with intestinal cells and maintained the integrity of the GBB (Bajaj et al., 2015). Several *Lactobacillus* spp. induce gene-regulation pathways that lead to upregulation of IL-1β, resulting in the transcription of genes involved in B-cell maturation and lymphogenesis, which contributes toward enhanced barrier stability and function. *L. plantarum* regulated human epithelial TJ proteins *in vivo* and conferred protective effects against chemically induced disruption of the epithelial barrier in an *in vitro* model (Karczewski et al., 2010). Administration of *L. plantarum* into the duodenum of healthy human volunteers significantly increased ZO-1 and occludin in the vicinity of TJ structures (Karczewski et al., 2010). These results suggest that administration of *L. plantarum* can enhance the stability of TJ complexes in humans and may attenuate their disruption by cytokines, toxins, and pathogens.

The serine protease inhibitor (serpin) produced by *Bifidobacterium longum* subsp. *longum* NCC2705 interact directly with the host factors (Ivanov et al., 2006). Extracellular serpin is also produced by other bifidobacterial species, including *B. breve*, *Bifidobacterium dentium*, and *B. longum* subsp. *infantis*. Serpin inhibits pancreatic and neutrophil elastases (Ivanov et al., 2006). Neutrophils are recruited in the intestinal mucosa from the blood vessels by means of the secretion of inflammatory cytokines. Serpin produced by bifidobacterian act on enzymes directly involved in the inflammatory response and might thus mediate some of the anti-inflammatory effects reported for bifidobacteria (Ivanov et al., 2006).

Proteinaceous compounds secreted by probiotic strains of *L. plantarum*, *L. acidophilus*, *Lactobacillus casei*, and *Lactobacillus delbrueckii* subsp. *bulgaricus* stimulated the expression of the muc2 gene and increased the production of mucin by murine colonic epithelial cells (Caballero-Franco et al., 2007). Extracellular proteins produced by *L. rhamnosus* GG increased production of the heat-shock proteins HSP25 and HSP72 in murine colon cells (Tao et al., 2006). Protein p40, produced by *L. rhamnosus* GG, is homologous to an uncharacterized surface antigen of *L. casei* ATCC 334 (gi| 116493594) and protein p75 to a cell wall-associated hydrolase of strain ATCC 334 (gi| 116493849; Yan et al., 2007). Both these proteins induced the proliferation of murine colonic epithelial cells and reduced injuries to colonic cells caused by tumor necrosis factor alpha (TNF-a; Yan et al., 2007). Proteins p40 and p75 inhibited TNF-a-induced apoptosis in the KSRI2/2 MCE cell line (Yan et al., 2007) and attenuated the TER decrease induced by hydrogen peroxide. Concluded from these results, proteins p40 and p75 play an important role in cell proliferation, apoptosis, and maintenance of the mucosal barrier.

## Evidence in Support of Bacteriocins Crossing the GBB

Little is known about the cellular receptors responsible for the recognition of extracellular proteins (thus also bacteriocins) secreted by gut microbiota (Asong et al., 2009). Bacterial flagellins are recognized by Toll-like receptor 5 (TLR-5) and by the ICE protease-activating factor (IPAF; Gewirtz, 2006; Ren et al., 2006). A particular TLR may recognize more than one type of molecule, as in the case of TLR-2 recognizing different glycolipids and lipoproteins (Yan et al., 2007). The C-type lectin receptor (CLR) DC-specific ICAM-3-grabbing non-integrin (DC-SIGN) may be involved in the recognition of extracellular components produced by probiotic bacteria (Konstantinov et al., 2008). The CLRs on the surface of immune cells, such as dendritic cells (DCs) and macrophages, recognize carbohydrate patterns and thus also glycoproteins (Benz and Schmidt, 2002). Glycolipids produced by lactobacilli are recognized by intestinal receptors (Iwamori et al., 2009).

Bacteriocin gene clusters are widespread in the genomes of intestinal bacteria (Drissi et al., 2015). Several studies have shown that bacteriocins are produced in the GIT and protect the host against infection (Bron et al., 2004; Corr et al., 2007; Van Zyl, 2018). It is likely that bacteriocins interact with epithelial cells in the GIT and even cross the GBB. Support for this comes from studies conducted by Spadoni et al. (2015). The authors showed that molecules of 4 kDa can cross the GVB. Most bacteriocins are smaller than 7 kDa, suggesting that they are small enough to cross the GVB. Other properties, such as charge, hydrophobicity, and affinity to IECs and ECs also have to be taken into account. A recent study has shown that bacteriocins can indeed transverse epithelial (Caco-2) and endothelial (HUVECs) monolayers (Dreyer, 2018). Nisin A (3.35 kDa), plantaricin 423 (3.93 kDa), and bacST4SA (4.29 kDa), labeled with NHS-fluorescein, crossed the epithelial and ECs without changing the integrity of the monolayers or having a toxin effect (Dreyer, 2018). Although the exact mechanism for crossing IECs and ECs was not examined, this study (albeit *in vitro*) provides evidence that bacteriocins can cross the GBB. Crossing of these peptides without eliciting a cytotoxic reaction suggests that they were transported paracellular. However, migration of cationic peptides via transcytosis (i.e., transcellular) cannot be ruled out and it may be the way larger bacteriocins cross the GBB. Transcytosis has been demonstrated for cell-penetrating peptides and bacterial toxins (e.g., botulinum toxin and cholera toxin). While bacteriocins have not been shown to cross the GBB they have been used in studies where intravenous injections resulted in treatment of intraperitoneal/subcutaneous infections (Goldstein et al., 1998; Castiglione et al., 2007; Jabés et al., 2011). Although this is different from the GBB, it supports the idea that bacteriocins are capable of crossing endothelial barriers.

Irrespective of the method bacteriocins may transverse the GBB, other challenges must also be overcome. Intestinal conditions are not favorable for long-term bacteriocin survival, with proteases and the mucus layer being the two most significant barriers. The mucus layer is capable of binding peptides; however, continuous production of bacteriocins may result in saturation, with some of the peptides reaching the GBB. Another possibility is bacteriocin production near the GBB, minimizing contact with the mucus layer. Proteases can also degrade bacteriocins before they reach the GBB. This may also be overcome by continuous production of bacteriocins by cells colonized close to the GBB. Of all sections in the GIT, bacteriocins are more likely to survive conditions in the colon where protease levels are lower.

Based on the few studies thus far reported, bacteriocins (maybe only a select few) do have the ability to cross the GBB. However, more research is required to determine when and how bacteriocins cross epithelial cells. Research should focus on the mechanisms bacteriocins use to migrate over IECs and ECs and whether specific receptors are involved. Furthermore, this should be translated into the complex *in vivo* environment of the GIT, keeping in mind that gut epithelial cells is not the only barrier. Once in the blood stream, bacteriocins may cross the blood-brain barrier. Little is known about the effect bacteriocins have once they cross these barriers.

## Potential Effects of Bacteriocins Across the GBB

Although bacteriocins are generally non-toxic and considered safe, exceptions do exist, e.g., cytolysin produced by enterococci, which has wide-spread cytotoxic activity (Cox et al., 2005). In most cases, cytotoxicity has only been noted at levels much higher than the minimal inhibitory concentration (MIC) required to inhibit food spoiling microorganisms (Lohans and Vederas, 2012). A crude extract of antimicrobial peptides isolated from *L. plantarum* LR/14 delayed the life cycle of *Drosophila melanogaster* when administered at 10 mg/mL (Gupta et al., 2014). The antimicrobial peptide P40, produced by *Bacillus licheniformis* P40, was cytotoxic to VERO cells when tested *in vitro* (Vaucher et al., 2010). A few bacteriocins displayed activity against sperm and tumor cells (Reddy et al., 2004). This is not that surprising, as bacteriocins may adhere to other negatively charged molecules or non-bacterial lipophilic surfaces. In a physiological environment this may cause a decrease in bio-availability. Bacteriocins can bind to blood cells and plasma proteins (Van Heel et al., 2011; Dreyer, 2018). Size and biochemical properties of peptides administered orally may also influence uptake and stability in the GIT (Cavera et al., 2015).

Bacteriocins are membrane active cationic peptides, and may thus also have an effect on mammalian cell membranes. Cinnamycin and duramycin bind phosphatidylethanolamine (PE), a substrate for phospholipase A2 and involved in inflammatory responses (e.g., vascular inflammation). The sequestering of PE by cinnamycin and duramycin may thus result in immune modulation through the indirect inactivation of phospholipase A2. Furthermore, by binding PE, the peptides may be deposited in cellular membranes, thereby potentially changing biophysical membrane properties. These changes can lead to altered ion channel functioning. In the case of duramycin, this characteristic is exploited for the potential treatment of cystic fibrosis. Minimal cytotoxic effects of bacteriocins against human cell lines have been reported (Murinda et al., 2003; Sand et al., 2010; Begde et al., 2011; Kindrachuk et al., 2013; Dreyer, 2018). Given the concentration of bacteriocin required to induce significant cytotoxicity, these levels would most likely not be present as a result of bacteriocins crossing the GBB. This, however, does not discount the possibility of bacteriocins accumulating in organs such as the liver and causing membrane damage.

If permeability is severely changed, gut microbiota may enter the blood stream and cause bacteremia. Certain pathogens can disrupt intracellular junctions by interacting with cell receptors. Enteric pathogens often gain access to the body by altering the structure and function of TJs to increase permeability of the barrier via the secretion of proteases, which can cleave TJ proteins or by altering the cytoskeleton (Berkes et al., 2003). Inflammatory cytokines such as TNFα and IFNγ, which are induced during infection and in IBD, increase intestinal permeability in general, although single inflammatory models yielded different results (Corridoni et al., 2012). Probiotics and commensal microbiota can reverse such inflammatory dysfunctions in human IECs. This is done by improving barrier functions or by inhibition of pathogen adherence (Resta-Lenert and Barrett, 2006; Moorthy et al., 2009; Qin et al., 2009; Anderson et al., 2010). Synergistic effects between sIgA and probiotics have been published (Mathias et al., 2010).

The lantibiotics gallidermin, Pep5, and nisin induce the release of multiple chemokines at levels similar to that of the human cationic antimicrobial peptide LL-37, with nisin seemingly able to activate multiple signaling pathways, including ERK/MAPK, PKC, and PKA (Kindrachuk et al., 2013). Nisin administered prophylactically to mice confers protection to mice challenged with Gram-positive (*S. aureus*) and Gram-negative (*S. enterica* and *E. coli*) bacteria. This is significant keeping in mind that nisin is ineffective against Gram-negative bacteria, suggesting that nisins’ interaction with the host’s immune response provides a selective advantage. At high concentrations, nisin activates neutrophils, resulting in formation of neutrophil extracellular traps (Begde et al., 2011). Neutrophil extracellular traps are known for trapping and killing bacteria (Zawrotniak and Rapala-Kozik, 2013). Furthermore, loci harboring genes involved in bacteriocin production and secretion modulate the immune response of dendritic and peripheral blood mononuclear cells (Meijerink et al., 2010; van Hemert et al., 2010). By enhancing the hosts’ immune system, bacteriocins indirectly provide protection against infectious microbial agents. These effects are not that surprising as host cationic defense peptides also have immune modulatory effects.

**FIGURE 2:**
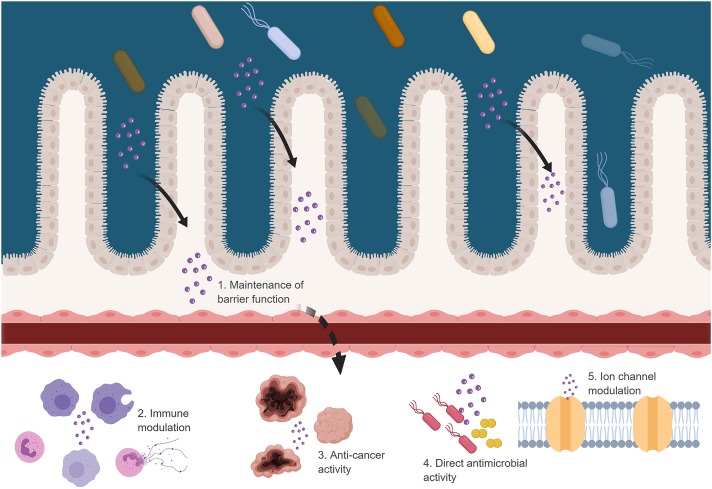
Possible effects of bacteriocins when crossing the GBB. Figure created in biorender (http://biorender.io).

Several studies have shown that some bacteriocins have anticancer properties (Kaur and Kaur, 2015). Bacteriocins have a higher affinity for cancer cells due to the general negative charge of cancer cells. Treatment of head and neck squamous cell carcinoma (HNSCC) cells with nisin induced DNA fragmentation and apoptosis on three different cancer cell lines (Joo et al., 2012; Kamarajan et al., 2015). Apoptosis in NHSCC cells, caused by nisin, is associated with calcium influx and upregulation of CHAC1 (cation transport regulator and apoptosis mediator; Joo et al., 2012). In another study, the size of tumors in mice with oral cancer was reduced when treated with nisin (Joo et al., 2012). The authors concluded that the selective action of nisin was due to structural differences in the composition of the plasma membranes between HNSCC cells and primary keratinocytes. The class IIc human defensins-like bacteriocin, laterosporulin 10, displays cytotoxic effects against several cell lines and causes necrotic and apoptotic cell death at high and low concentrations, respectively. At high concentrations (10 μM), more than 95% of normal prostate epithelial cells remained viable, whereas 80% of cancer cells lost their viability. As with cytotoxicity against normal cells, the concentrations used to be effective against cancerous cells may be higher than the levels crossing the GBB. However, the higher affinity for cancerous cells may result in bacteriocins targeting these cells. Immune priming by bacteriocins may also assist in the elimination of cancer cells. The possibility of bacteriocins crossing the GBB is intriguing and from the literature, it is clear that they are capable of effecting the host if they do cross (**Figure 2**). However, if they do cross and if they exert an effect requires further investigation.

## Conclusion

The abundance and diversity of bacteriocins make them ideal candidates for use in the treatment and prevention of infections. In addition to high natural diversity, the bio-engineering of bacteriocins and improvements in production processes allow us to tailor bacteriocins so that they fit specific needs. Non-antimicrobial properties of bacteriocins, such as immune modulation, also introduce new applications. The most pertinent question to answer is to what extent these bacteriocins are able to cross the GBB to achieve these beneficial effects systemically. Approval by medical control councils for using a newly developed drug is a slow and tedious process and involves a number of safety tests and clinical trials. Natural antimicrobial peptides and bacteriocins adhere to the same rules and regulations. To establish the safety of bacteriocins, a number of tests will have to be conducted. These include cytotoxicity studies incorporating eukaryotic cell lines, the ability to induce apoptosis, inhibit cellular growth, alter metabolic functions, and lyze red blood cells (hemolytic activity). Further tests may include the development of resistance to antimicrobial activity at therapeutic levels, effect on the host’s immune system, and the development of allergies.

## Author Contributions

All authors contributed equally to the manuscript.

## Conflict of Interest Statement

The authors declare that the research was conducted in the absence of any commercial or financial relationships that could be construed as a potential conflict of interest.
