# Corrigendum: A Review: The Fate of Bacteriocins in the Human Gastro-Intestinal Tract: Do They Cross the Gut–Blood Barrier?

**DOI:** 10.3389/fmicb.2018.02938

**Published:** 2018-11-30

**Authors:** Leon M. T. Dicks, Leané Dreyer, Carine Smith, Anton D. van Staden

**Affiliations:** ^1^Department of Microbiology, Stellenbosch University, Stellenbosch, South Africa; ^2^Department of Physiological Sciences, Stellenbosch University, Stellenbosch, South Africa

**Keywords:** bacteriocins, antibiotics, microbiota, gut–blood barrier, probiotics

In the original article, there was an error: Incorrect presentation of bacteriocin classification schemes.

A correction has been made to the Introduction, paragraph one:

Bacteriocins are ribosomally synthesized peptides with antibacterial activity (Cavera et al., 2015). Bacteriocins can be post-translationally modified (PTM) or non-modified and are grouped into different classes (Cotter et al., 2005; Heng et al., 2006; Arnison et al., 2013; Alvarez-Sieiro et al., 2016). For example, lantibiotics are modified bacteriocins and are grouped in class I, they are membrane-active peptides with thioether-containing amino acids lanthionine and β-methyllanthionine. According to the classification scheme proposed by Heng et al. (2006), class I is subdivided into three types of lantibiotics (types A–C). Linear lantibiotics were classified as type A, globular lantibiotics as type B, and multi-component lantibiotics, requiring two or more modified peptides for bioactivity as type C. Subsequent classification schemes for class I have been updated, including changes to the classification of lantibiotics and inclusion of other PTM bacteriocins (Arnison et al., 2013; Alvarez-Sieiro et al., 2016). An extensive review by Arnison et al. (2013) expanded on the nomenclature and classification of modified bacteriocins with the proposed recommendations changing and expanding on previous classification schemes. For example, lanthipeptides (including lantibiotics) are separated into four classes based on modification machinery and using this classification system for lanthipeptides each class can further be grouped based on amino acid sequences of the modified peptides (Cotter et al., 2005; Arnison et al., 2013; Van Staden, 2015). Additionally, the review by Arnison et al. (2013) also expands on the classification and nomenclature of non-lantibiotic/lanthipeptide modified peptides. As reviewed by Alvarez-Sieiro et al. (2016), the unmodified, membrane active, heat stable bacteriocins are grouped in class II, with four subclasses based on structural differences and their mode of action. Anti-listeria, pediocin-like peptides, are grouped in subclass IIa and bacteriocins that require two or more peptides for activity in subclass IIb. Leaderless bacteriocins, without an N-terminal leader peptide, are grouped in class IIc and unmodified bacteriocins that are not pediocin-like or multi-component bacteriocins are grouped in subclass IId. Large heat liable bacteriocins are grouped in class III, subdivided into IIIa (bacteriolysins) and IIIb (non-lytic proteins).

Classification schemes for bacteriocins are constantly evolving to accommodate the increase in complexity and diversity of these peptides. Furthermore, with increased understanding of how bacteriocins function and identification of novel bacteriocins the systems in place to group them will need to adapt and change accordingly.

The authors apologize for this error and state that this does not change the scientific conclusions of the article in any way. The original article has been updated.

## Conflict of interest statement

The authors declare that the research was conducted in the absence of any commercial or financial relationships that could be construed as a potential conflict of interest.
